# Ten‐year clinical outcomes in patients with intermediate coronary stenosis according to the combined culprit lesion

**DOI:** 10.1002/clc.23668

**Published:** 2021-06-16

**Authors:** Yong Kyun Kim, Chae Won Jang, Soon Ho Kwon, Jae Hoon Kim, Amir Lerman, Jang‐Ho Bae

**Affiliations:** ^1^ Division of Cardiology, Department of Internal Medicine Konyang University Hospital Daejeon South Korea; ^2^ Department of Cardiovascular Disease Mayo Clinic Rochester Minnesota USA

**Keywords:** coronary artery stenosis, intermediate coronary lesion, prognosis

## Abstract

**Background:**

We assessed the long‐term clinical outcomes of an intermediate lesion (IL) according to the presence of a combined culprit lesion (CCL).

**Hypothesis:**

Long‐term clinical outcomes of IL may be affected by the presence of a CCL.

**Methods:**

Angiographic findings (*n* = 1096) and medical chart were reviewed. Patients with IL were divided into two groups: IL without CCL group (*n* = 383, 64.5%) and IL with CCL group (*n* = 211, 35.5%).

**Results:**

The major adverse cardiovascular events (MACE) in the IL with CCL group were significantly higher than those in the IL without CCL group (death: 12.3% vs. 7.0%, myocardial infarction: 3.3%vs. 0.5%, stroke: 6.6% vs. 2.6%, and revascularization [RVSC]: 25.1% vs. 7.6%) during a mean follow up period of 118.4 ± 5.5 months. IL related RVSC rate in the IL with CCL group was higher than that in the IL without CCL group (5.7% vs. 2.1%, p = 0.020). RVSC rate related to IL in total subjects was lower than that related to stented lesion (3.4% vs. 6.4%). The important predictors of total MACE in total subjects were the presence of CCL, IL percent diameter stenosis, hypertension, history of percutaneous coronary intervention, blood glucose and ejection fraction. The predictors of IL related RVSC were IL percent diameter stenosis and IL located in the right coronary artery.

**Conclusion:**

10‐year clinical outcomes of an IL (especially IL without CCL) were better than those of stented lesions. This study suggests that the IL can be safely followed up in sites that do not have ability to assess functional study.

## 1 INTRODUCTION

The prevalence of an intermediate lesion (IL) is relatively high, ranging from 39.4% to 79.3%.^1^, ^2^, ^3^, ^4^ Previous studies have evaluated the value of functional status assessment and prognostic factors of the intermediate lesion.^5^, ^6^, ^7^ Fractional flow reserve (FFR) index is considered as a gold standard to assess whether the intermediate stenosis is responsible for inducible ischemia and whether the patient would benefit from revascularization (RVSC). Therefore, current guidelines recommend FFR measurements for the assessment of the IL.^8^, ^9^ The DEFER (deferral versus performance of balloon angioplasty in patients without documented ischemia) and FAME (fractional flow reserve versus angiography for multi‐vessel evaluation) studies showed better 5‐year and 2‐year clinical outcomes in patients treated by FFR guided therapeutic decision than those treated by angiography guided decision.^1^, ^2^, ^6^, ^10^

However, recent analysis of the FAME study showed that the improved clinical outcomes of FFR‐guided percutaneous coronary intervention (PCI) group, which were observed in the first 2 years after the index procedure, did not persist until 5 years.^11^ And 65% (402/620) of the intermediate lesions showed FFR > 0.80 in other study.^6^ Moreover, functional stenosis severity of non‐culprit lesions is frequently overestimated.^12^ And the utilization of FFR is increasing, however, the many cardiac laboratories still making clinical decision based on coronary angiography alone due to the equipment availability, reimbursement policies, and other financial considerations.^13^, ^14^

According to many studies, conservative medical treatment of IL might be safe and justified; therefore, performing PCI may be safely deferred in patients with such lesions.^15^, ^16^, ^17^ We previously reported that 10‐year clinical outcomes of the IL was favorable compared with those of the significantly stenotic culprit lesion; however long‐term outcomes of deferred IL based on angiography alone remain unclear^18^ Our previous study was performed in patients with multi‐vessel disease including an IL. Therefore long‐term outcomes of the IL without combined culprit lesion are unclear. Furthermore, one needs to consider the risk and benefit of PCI in those lesions such as resource use (FFR, PCI), procedure related problems and antiplatelet related problems, et al.^19^, ^20^, ^21^

The objectives of this study were to find the real world very long‐term (10 years) clinical outcomes of an angiographically intermediate lesion (IL), to find the difference of the clinical outcomes of an IL according to the combined culprit lesion (CCL) and to find predictors of the adverse clinical outcomes in a large number of patients with IL.

## 2 METHODS

### 2.1 Study population

This study was a non‐randomized, retrospective, single center study. We analyzed the medical records of 1096 patients who underwent coronary angiogram (CAG) in Konyang University Hospital, Daejeon, South Korea between January 2008 and December 2008. We reviewed patient demographics and laboratory and angiographic findings. IL was defined as 30% to 70% angiographic stenosis visually estimated from baseline CAG.^22^ Two cardiologists (KYK and JCW) reviewed and determined angiographic stenosis. All disagreements between two cardiologists were resolved by a senior cardiologist (BJH). We excluded 502 patients without IL. Finally, a total of 594 patients were enrolled in this study. Enrolled patients were followed‐up over a pre‐defined follow‐up period to be 10 years. The study subjects were divided into 2 groups according to the angiographically presence or absence of CCL in other major epicardial coronary arteries; IL without CCL group and IL with CCL group. The IL without CCL group included patients who had IL without other significant CCL (angiographic percent stenosis ≥70%), which was mostly treated with PCI.

The study was approved by the Institutional Review Board of Konyang University Hospital and was performed in accordance with the ethical guidelines of the 1975 Declaration of Helsinki.

### 2.2 Study endpoint

The primary outcome of this study was the occurrence of major adverse cardiovascular event (MACE), which was defined as all‐cause death, myocardial infarction (MI), stroke, and revascularization (RVSC; defined as PCI or coronary artery bypass grafting involving the target lesion). Adverse events were reviewed and adjudicated by at least two independent investigators blinded to the study groups. MI was defined as chest pain with or without a ST‐segment elevation ≥2 mm in ≥2 contiguous precordial leads, or ≥ 1 mm in ≥2 limb leads, or a new left bundle branch block on electrocardiogram, and elevation of cardiac enzymes at least 3 times the upper limit of the normal range. Stroke was defined as neurological symptoms associated with radiologic findings based on computed tomography or magnetic resonance imaging. The simultaneous or sequential occurrence of two or more MACE was counted as one incidence of MACE, and time‐to‐event duration was defined as the duration between enrollment and the first event.

### 2.3 Statistical analysis

Continuous variables are presented as mean ± standard deviation and categorical variables are presented as numbers and percentages. The Chi‐square test or Fisher's exact test was performed to analyze categorical variables, and an independent *t* test was performed to analyze continuous variables in the univariate analysis. The Kaplan–Meier method was used to assess cumulative event rates. Multivariate Cox‐proportional hazard analysis was performed to investigate the independent predictors of MACE, RVSC, and IL related RVSC. Clinically relevant coronary artery disease risk factors and angiographic finding variables that showed significant relationships with MACE and RVSC in the univariate analysis (p < 0.10) were included into the multivariate analysis.

All statistical analyses were conducted using the SPSS (version 18.0), and a p‐value <0.05 was considered statistically significant.

## 3 RESULT

### 3.1 Patient demographics

The study population including 330 (55.6%) men had a mean age of 64.4 ± 10.8 years (Table 1). The mean follow up duration in the IL without CCL group and IL with CCL group was 118.4 ± 5.3 months and 118.5 ± 5.8 months (p = 0.901), respectively. The IL with CCL group had more patients with acute coronary syndrome (ACS) than the IL without CCL group (36.5% vs. 5.0%, p < 0.001). Compared with the IL without CCL group, the IL with CCL group had significantly higher level of total cholesterol (194.2 ± 58.2 mg/dl vs. 183.6 ± 48.0 mg/dl, p = 0.019), LDL cholesterol (121.5 ± 35.5 mg/dl vs. 115.1 ± 35.3 mg/dl, p = 0.038) and glucose (157.3 ± 80.1 mg/dl vs. 138.1 ± 59.5 mg/dl, p = 0.002), and lower ejection fraction (65.2 ± 11.6% vs. 67.7 ± 9.9%, p = 0.008). The IL without CCL group had higher number of patients with a history of PCI (21.4% vs. 20.9%, p = 0.035) (Table 1). There was no other significant difference in other demographics and laboratory findings between the two groups.

**TABLE 1:** Patients demographic and lesion characteristics

Variables	IL without CCL	IL with CCL	Total	p‐value
Patient number, *n* (%)	383 (64.5)	211 (35.5)	594 (100)	
FU loss, *n* (%)	138 (36.0)	57 (27.0)	195 (32.8)	0.008
FU duration, month				
Total subjects	89.8 ± 40.9	97.0 ± 37.7	92.4 ± 39.9	0.032
Those with FU	118.4 ± 5.3	118.5 ± 5.8	118.4 ± 5.5	0.901
Those with FU loss	39.1 ± 23.6	39.0 ± 23.6	39.0 ± 23.5	0.982
Age, years	63.8 ± 10.2	65.5 ± 11.7	64.4 ± 10.8	0.085
Male, *n* (%)	204 (53.3)	126 (59.7)	330 (55.6)	0.130
Hypertension, *n* (%)	204 (53.3)	117 (56.8)	321 (54.5)	0.412
Diabetes mellitus, *n* (%)	91 (23.8)	60 (29.1)	151 (25.6)	0.155
Smoking, *n* (%)	79 (20.6)	53 (25.7)	132 (22.4)	0.288
Diagnosis, *n* (%)				<0.000
Stable angina	357 (93.2)	134 (63.5)	491 (82.7)	
ACS	19 (5.0)	77 (36.5)	96 (16.2)	
Others	7 (1.8)	0 (0.0)	7 (1.3)	
Lipid profile				
Total cholesterol, mg/dl	183.6 ± 48.0	194.2 ± 58.2	187.4 ± 52.1	0.019
Triglyceride, mg/dl	166.0 ± 115.2	193.0 ± 195.6	175.8 ± 149.9	0.070
HDL cholesterol, mg/dl	49.4 ± 36.1	48.9 ± 36.1	49.2 ± 36.1	0.868
LDL cholesterol, mg/dl	115.1 ± 35.3	121.5 ± 35.5	117.5 ± 35.5	0.038
Creatinine, mg/dl	1.09 ± 0.73	1.28 ± 1.47	1.16 ± 1.06	0.071
Glucose, mg/dl	138.1 ± 59.5	157.3 ± 80.1	144.9 ± 68.1	0.002
Hs C‐reactive protein, mg/L	1.73 ± 10.44	4.54 ± 21.46	2.73 ± 15.34	0.082
Ejection fraction, %	67.7 ± 9.9	65.2 ± 11.6	66.9 ± 10.6	0.008
Previous PCI, *n* (%)	82 (21.4)	44 (20.9)	126 (21.2)	0.035
Previous CABG, *n* (%)	0 (0.0)	4 (1.9)	4 (0.7)	0.035
IL, *n* (%)	484 (62.5)	291 (37.5)	775 (100)	0.019
Location				
LAD, *n* (%)	254 (66.3)	119 (56.4)	373 (62.8)	0.017
LCX, *n* (%)	103 (26.9)	66 (31.3)	169 (28.5)	0.257
RCA, *n* (%)	127 (33.2)	106 (50.2)	233 (39.2)	0.000
Percent diameter stenosis (%)	42.4 ± 13.6	54.4 ± 13.2	46.6 ± 14.6	0.000
CCL, *n* (%)		293 (100)		
Location				
LAD, *n* (%)	NA	122 (57.8)		
LCX, *n* (%)	NA	86 (40.8)		
RCA, *n* (%)	NA	85 (14.3)		
Percent diameter stenosis (%)		91.3 ± 7.9		
Treatment for CCL (patient level)				
PCI + optimal medication	NA	174 (82.5)		
Optimal medication only	NA	37 (17.5)		

Abbreviations: ACS, acute coronary syndrome; CABG, coronary artery bypass graft; CCL, combined culprit lesion; FU, follow up; HDL, high density lipoprotein; Hs C –reactive protein, hypersensitivity C‐reactive protein; IL, intermediate lesion; LAD, left anterior descending coronary artery; LCX, left circumflex coronary artery; LDL, low density lipoprotein; NA, not applicable; PCI, percutaneous coronary intervention; RCA, right coronary artery.

### 3.2 Angiographic findings

This study included 775 ILs from 594 patients. There were total 293 CCLs in 594 patients at baseline. The IL without CCL group comprised of 484 ILs in 383 patients, whereas the IL with CCL group consisted of 291 ILs in 211 patients (Table 1).

IL was most frequently located in the left anterior descending artery (LAD, *n* = 373, 62.8%) followed by the right coronary artery (RCA, *n* = 233, 39.2%) and left circumflex artery (LCX, *n* = 169, 28.5%). The ILs in the IL without CCL group were located more frequently in the LAD (*n* = 254, 66.3% vs. *n* = 119, 56.4%, p = 0.017) and less frequently located in the RCA (*n* = 127, 33.2% vs. *n* = 106, 50.2%, p < 0.001) than those in the IL with CCL group. The IL with CCL group had a significantly higher angiographic percent diameter stenosis (54.4 ± 13.2% vs. 42.4 ± 13.6%, p < 0.001) than the IL without CCL group.

In the IL with CCL group, culprit lesions were located most frequently in the LAD (*n* = 122, 57.8%), followed by the LCX (*n* = 86, 40.8%) and RCA (*n* = 85, 14.3%). The mean angiographic percent diameter stenosis of CCL was 91.3 ± 7.9%.

The majority of the CCLs (82.5%) were treated with PCI and optimal medication (Table 1).

### 3.3 Clinical outcomes

During a mean follow‐up period of 118.4 ± 5.5 months, 140 MACE occurred, including 61 events in the IL without CCL group and 79 events in the IL with CCL group (Table 2). IL with CCL group had a higher incidence of MACE than the IL without CCL group (death: 12.3% vs. 7.0%, p = 0.031, MI: 3.3% vs. 0.5%, p = 0.012, stroke: 6.6% vs. 2.6%, P = 0.017, and RVSC: 25.1% vs. 7.6%, p < 0.001).

**TABLE 2:** Comparison of MACE between 2 groups

	IL without CCL	IL with CCL	All events	p‐value
MACE, *n* (%)	61 (15.9)	79 (37.4)	140 (23.6)	0.000
Death, *n* (%)	27 (7.0)	26 (12.3)	53 (8.9)	0.031
MI, *n* (%)	2 (0.5)	7 (3.3)	9 (1.5)	0.012
Stroke, *n* (%)	10 (2.6)	14 (6.6)	24 (4.0)	0.017
Revascularization, *n* (%)	29 (7.6)	53 (25.1)	82 (13.8)	0.000
New lesion, *n* (%)	9 (2.3)	12 (5.7)	21 (3.5)	0.035
CCL, *n* (%)	NA	4 (1.9)	4 (0.7)	0.016
IL, *n* (%)	8 (2.1)	12 (5.7)	20 (3.4)	0.020
Stented lesion, *n* (%)	12 (3.1)	26 (12.3)	38 (6.4)	0.000

Abbreviations: CCL, combined culprit lesion; IL, Intermediate lesion; MACE, major adverse cardiovascular event; MI, myocardial infarction; NA, not applicable.

The most common cause of RVSC was in‐stent restenosis (ISR, 6.4% during 10 years of follow up) in both the IL without CCL group and IL with CCL group, but RVSC rate due to ISR was significantly higher in the IL with CCL group than in the IL without CCL group (*n* = 26, 12.3% vs. *n* = 12, 3.1%, p < 0.001) (Table 2).

Both the rates of RVSC related to IL (Figure S1A) and RVSC related to a new lesion, which initially appeared normal or showed less than 30% stenosis on baseline angiogram, (Figure S1B) were also higher in the IL with CCL group than those in the IL without CCL group (IL related RVSC: 5.7% vs. 2.1%, p = 0.020; new lesion related RVSC: 5.7% vs. 2.3%, p = 0.035) during 10 years of follow up.

In the IL with CCL group, the rate of RVSC due to CCLs, which was initially treated with optimal medication, was 1.9% during 10 years of follow up (Table 2).

**FIGURE 1:**
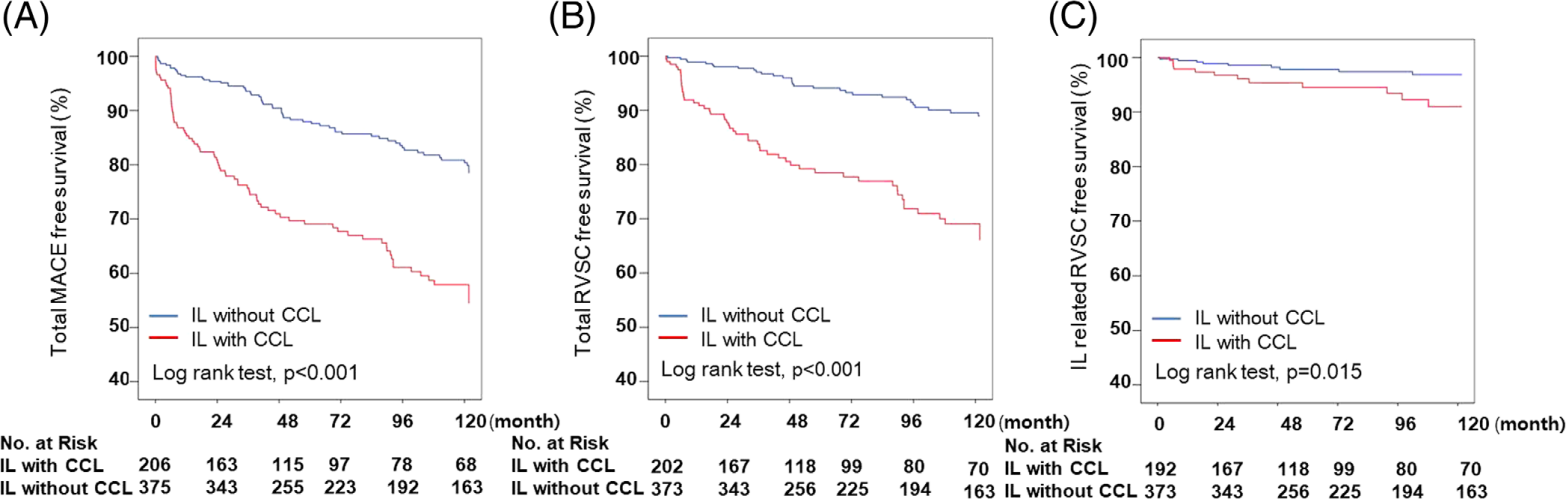
(A; left, B; middle, C; right). Kaplan–Meier curves according to study group for survival free from (A) total MACE, (B) total RVSC and (C) IL related RVSC during 10‐years follow up period. CCL, combined culprit lesion; IL, intermediate lesion; MACE, major adverse cardiovascular event; RVSC, revascularization

The Kaplan–Meier curves for the primary endpoint during 10 years of follow up are shown in Figure 1. There was significant difference in all event‐free survival between the IL without CCL group and IL with CCL group. As presented in Figure 1, the 10‐year cumulative total MACE–free survival was significantly higher in the IL without CCL group compared with that in the IL with CCL group (78.4% vs. 54.4%, p < 0.001) (Figure 1.A). The total RVSC–free survival in the IL without CCL group was also significantly higher than that in the IL with CCL group (89.0% vs. 66%, p < 0.001) (Figure 1.B). IL related RVSC–free survival in the IL without CCL group was higher than that in the IL with CCL group (96.9% vs. 91.0%, p = 0.015) (Figure 1C).

### 3.4 Predictors of MACE and RVSC

Adjusted multivariate Cox proportional hazard analysis revealed CCL (hazard ratio [HR] 2.107, 95% confidence interval [CI] 1.449–3.063, p < 0.001), IL percent diameter stenosis (HR 1.023, 95% CI 1.010–1.037, p = 0.001), hypertension (HR 1.486, 95% CI 1.037–2.131, p = 0.031), history of PCI (HR 2.281, 95% CI 1.584–3.285, p < 0.001), blood glucose level (HR 1.003, 95% CI 1.001–1.005, p = 0.003), and ejection fraction (HR 0.980, 95% CI 0.964–0.995, p = 0.010) as the predictors of total MACE (Table 3).

**TABLE 3:** Multivariate cox‐proportional hazard analysis for total MACE/RVSC and Intermediate lesion related RVSC

Total MACE						
	Unadjusted model			Adjusted model		
Variables	HR	95% CI	p value	HR	95% CI	p value
ACS	2.218	1.517–3.243	0.000			
CCL	2.764	1.978–3.861	0.000	2.107	1.449–3.063	0.000
IL percent diameter stenosis	1.037	1.025–1.049	0.000	1.023	1.010–1.037	0.001
Gender (male)	1.544	1.089–2.189	0.015			
HTN	1.425	1.009–2.013	0.044	1.486	1.037–2.131	0.031
Previous PCI	2.520	1.792–3.544	0.000	2.281	1.584–3.285	0.000
Glucose	1.004	1.002–1.005	0.000	1.003	1.001–1.005	0.003
Ejection fraction	0.976	0.962–0.990	0.001	0.980	0.964–0.995	0.010

Total RVSC						
	Unadjusted model			Adjusted model		
Variables	HR	95% CI	p value	HR	95% CI	p value
ACS	2.186	1.331–3.591	0.002			
CCL	3.863	2.456–6.076	0.000	3.515	2.162–5.714	0.000
IL percent diameter stenosis	1.040	1.024–1.056	0.000	1.023	1.005–1.041	0.011
IL in the RCA	1.854	1.202–2.860	0.005	1.622	1.042–2.525	0.032
Gender (male)	1.853	1.158–2.965	0.010			
Previous PCI	5.071	3.265–7.875	0.000	5.740	3.662–8.996	0.000

Intermediate lesion related RVSC						
	Unadjusted model			Adjusted model		
Variables	HR	95% CI	p value	HR	95% CI	p value
ACS	3.143	1.268–7.794	0.013			
IL percent diameter stenosis	1.054	1.021–1.089	0.001	1.054	1.021–1.089	0.001
IL in the RCA	2.538	1.037–6.210	0.041	2.111	0.889–5.011	0.090

Abbreviations: ACS, acute coronary syndrome; CCL, combined culprit lesion; CI, confidence interval; HR, hazard ratio; HTN, hypertension; IL, intermediate lesion; MACE, major adverse cardiovascular event; PCI, percutaneous coronary intervention; RCA, right coronary artery; RVSC, revascularization.

The predictors of total RVSC were CCL (HR 3.515, 95% CI 2.162–5.714, p < 0.001), IL percent diameter stenosis (HR 1.023, 95% CI 1.005–1.041, p = 0.011), IL located in the RCA (HR 1.622, 95% CI 1.042–2.525, p = 0.032), and history of PCI (HR 5.740, 95% CI 3.662–8.996, p < 0.001) (Table 3).

When we considered only IL related RVSC, IL percent diameter stenosis (HR 1.054, 95% CI 1.021–1.089, p = 0.001) and IL located in the RCA (HR 2.111, 95% CI 0.889–5.011, p = 0.090) were the predictors of IL related RVSC after adjustment (Table 3).

Receiver operating characteristic (ROC) curve analysis (Figure S2) was used to determine the performance of IL percent diameter stenosis to predict total MACE and IL related RVSC. The area under the ROC curve was 0.67 and 0.70, respectively. At a cutoff value of IL percent diameter stenosis of 47.5%, the sensitivity and specificity for predicting total MACE were 73% and 57%, respectively (Figure S2A). Additionally, at a cutoff value of IL percent diameter stenosis of 47.5%, the sensitivity and specificity for predicting IL related RVSC were 86% and 52%, respectively (Figure S2B).

## 4 DISCUSSION

The main findings of this study are described below. The 10‐year cumulative incidence of MACE and RVSC was significantly lower in the IL without CCL group than IL with CCL group (MACE: 15.9% vs. 37.4%, p < 0.001, RVSC: 7.6% vs. 25.1%, p < 0.001).The rate of IL related RVSC was also significantly lower in the IL without CCL group compared with that in the IL with CCL group (2.1% vs. 5.7%, p = 0.020). The important predictors of total MACE were CCL, IL percent diameter stenosis, hypertension, history of PCI, blood glucose level, and ejection fraction. The significant predictors of all RVSC were CCL, IL percent diameter stenosis, IL located in the RCA, and history of PCI. The predictors of IL related RVSC were IL percent diameter stenosis and IL located in the RCA. Our results suggest that angiographically IL in patient without significant CCL would have a favorable clinical outcome with optimal medical treatment compared to that in patients with CCL. Furthermore, better clinical outcomes, especially that of RVSC, were observed in patients who had IL with CCL than in patients with stented lesion (5.7% vs. 12.3%) during 10 years follow up. The current study also showed that the presence of IL in patients with CCL was not associated with an increase in the incidence of MACE.

This study demonstrated that coronary angiographic degree of stenosis could be a predictor of MACE. However, there is some debate as to whether the severity of coronary stenosis is associated with future cardiovascular events.^3^, ^4^, ^23^ According to previous observational studies, RCA stenosis is frequently observed in patients with coronary artery disease and RCA disease progresses more rapidly; therefore, patients with RCA stenosis may be more vulnerable than those with LAD stenosis.^24^, ^25^

In line with these studies, our study showed that the IL located in the RCA was also an important predictor of RVSC.

In the present study, the rate of new lesion related RVSC was similar (3.5% vs. 3.4%) to that of IL related RVSC in all study subjects during 10 years of follow up. This result suggests that the rate of minimal lesion related RVSC is similar to that of IL related RVSC, although RVSC clearly has more favorable outcomes patients with minimal lesion and IL than patients with stented lesion (6.4%). It may also suggest that the presence of a CCL is not associated with disease progression. In particular, rate of RVSC due to ISR (12.3%) was almost twice that of RVSC due to new lesions (5.7%) or IL (5.7%) in the IL with CCL group during 10 years of follow‐up. Furthermore, the rate of IL related RVSC was significantly lower (2.1% vs. 5.7%, p = 0.020) in the IL without CCL group than that in the IL with CCL group. These results suggest more favorable outcomes in patients with IL and minimal lesion than in patients with stented lesions during 10 years of follow up. Additionally, the absence of CCL led to further improvement in outcomes. Therefore, treating IL with optimal medical therapy or monitoring IL without any further examination or intervention may be an alternative option. The results also suggest that these ILs are not a risk factor for MACE.

The 10‐year cumulative incidence of total MACE in this study was slightly higher (23.6% vs. 20.4%) than 3‐year cumulative incidence of total MACE in the PROSPECT trial. When we considered only the IL without CCL group, the incidence of total MACE in our study was much lower (15.9% vs. 20.4%) than that in the PROSPECT trial. The rate of RVSC due to stented lesion in the PROSPECT trial was comparable (10.9% vs. 12.3%) to that in the IL with CCL group of our study although the follow up duration was much longer (10 vs. 3 years) in our study than that in the PROSPECT trial. The rate of IL related RVSC was much higher (10.5% vs. 3.4%) in the PROSPECT trial than that in our study. The difference in the rate of RVSC between the two studies may be explained by the study population; the PROSPECT trial was conducted in ACS patients, whereas our study included mostly (82.7%)patients with stable angina. However, in the PROSPECT trial, the rates of RVSC due to culprit lesions and non‐culprit lesions were similar.

Currently, FFR and instantaneous wave‐free ratio (iFR) are used as a gold standard in making therapeutic decisions for IL.^8^, ^9^, ^26^ However, these technologies may not be available in all cardiac laboratories. In addition, the clinical application of functional study has been variable and remains underused.^13^, ^14^

The FAME and DEFER studies showed better clinical outcomes in patients treated by FFR guided therapeutic decision than those in patients treated by angiography guided decision. The FAME study showed that the benefit of PCI resulted from the routine measurement of FFR, which allowed the judicious use of stents.^2^, ^10^, ^11^ The DEFER study showed that PCI of a functionally non‐significant stenosis had no advantage and even resulted in more MI in the stented artery.^5^, ^27^ However, these two landmark studies were conducted in the era when the very long term clinical natural history of IL is unknown. In addition, there are several conflicting studies showing the questionable role of FFR in IL such as gray zone, overestimation and mostly negative FFR.^6^, ^12^ Meanwhile, it is important to consider the benefit of RVSC to overcome treatment related adverse event such as periprocedural complications, stent thrombosis, ISR and cost.

The presence and stenosis severity of CCL were the most significant and important prognostic factors of total MACE and RVSC. These findings are in accordance with those of previous studies reporting poorer prognosis of multi‐vessel coronary artery disease than that of single‐vessel disease.^28^, ^29^ The presence of CCL may imply a diffuse nature of coronary atherosclerosis or pathophysiological inflammatory process that involves the entire systematic cardiovascular system. Therefore, our study suggests that IL, with CCL, requires more careful surveillance and an optimal treatment strategy to prevent future cardiovascular events than IL without CCL.

## 5 STUDY LIMITATION

This was a retrospective study; therefore, the sources of selection bias in this study could not be excluded. Only 32.8% of patients were lost to follow up but the long‐term follow‐up partially compensated the limitation to evaluate the natural history of IL. Our study analyzed lesion stenotic severity but not lesion length, which is also an important geometric parameter. In addition, tandem lesions are considered as one lesion which is most stenosis. Although the entire stenotic segment contributes to the resistance, the most stenotic lesion is an important factor in limiting coronary flow.

This study was conducted over 10 years. The patients were followed up and managed by a dedicated doctor, usually in outpatients clinics. The patients were managed with conventional medication, which could be changed during the long follow up period. Another limitation of our study was that, the impact of medication on clinical outcomes was not analyzed. However, it was practically difficult for us to determine the impact of medications on clinical outcomes, especially in this long‐term retrospective clinical study, because of the variations in medications over time. These limitations require confirmation in a prospectively designed study.

## 6 CONCLUSION

Our study showed favorable very long‐term (10 years) outcomes of angiographically IL, especially IL without CCL and even better than those of stented lesions. Therefore, this study suggests that optimal medical treatment without any invasive work up or intervention may be a possible therapeutic option for IL in sites that do not have facility to assess FFR.

## CONFLICT OF INTEREST

The authors declare no potential conflict of interest.

## Supporting information

**Figure S1** xxxx

**Figure S2** xxxx

## Data Availability

Data sharing is not applicable as no datasets were generated or analyzed for this study.
