# Supplementary material for: Ten‐year clinical outcomes in patients with intermediate coronary stenosis according to the combined culprit lesion
Source: Clin Cardiol. 2021 Jun 16;44(8):1161–8. doi: 10.1002/clc.23668 (PMC8364722; doi:10.1002/clc.23668)
Supplement: Supplementary file 1 — Figure S1 xxxx [file CLC-44-1161-s002.pdf]

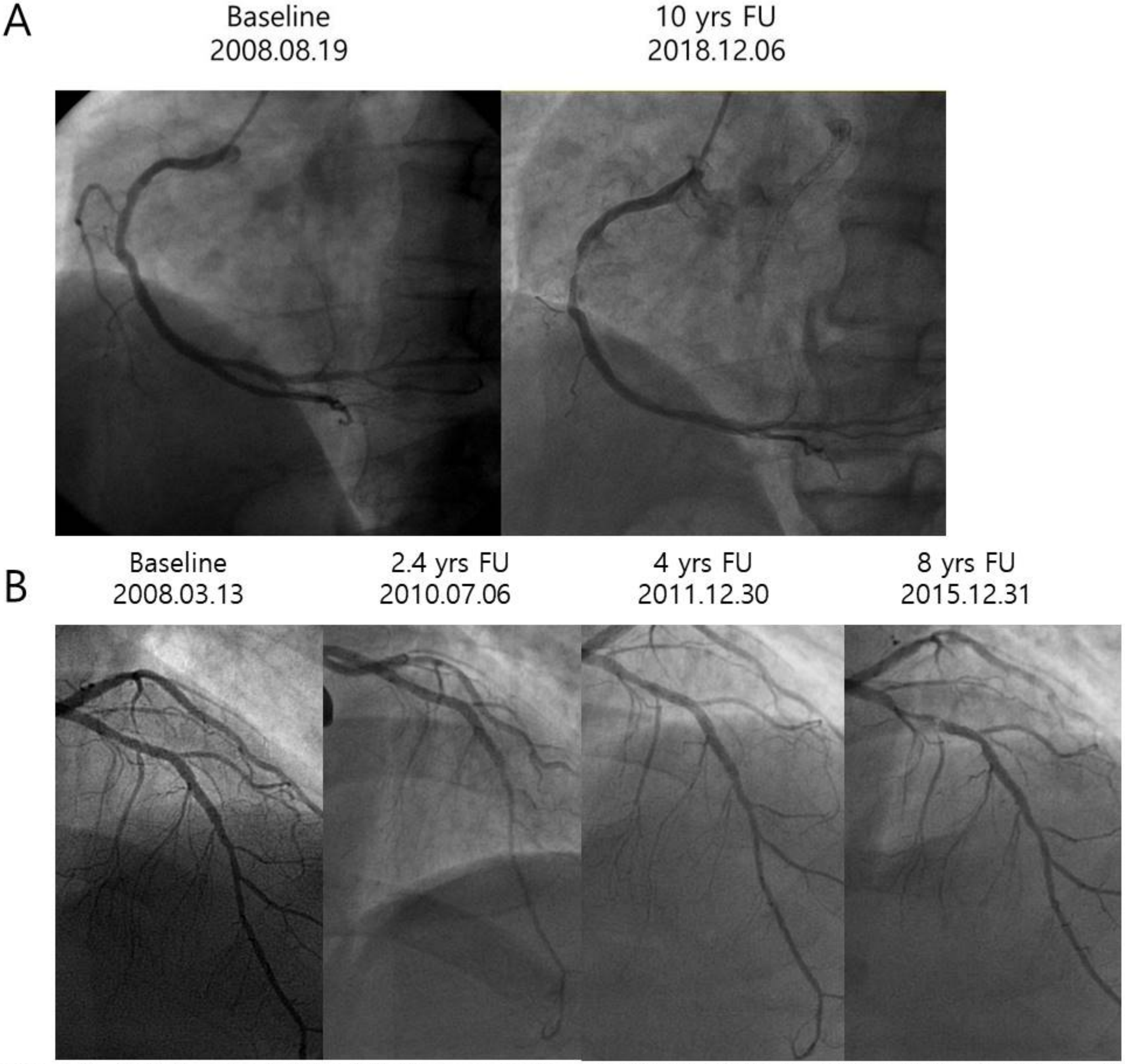

Supplementary figure 1. (A) IL at middle RCA lesion in a patient with IL with CCL group (LAD as a CCL was treated with stenting) progressed to severe stenosis with unstable angina and underwent revascularization at 10 years FU. (B) Minimal lesion at proximal LAD in a patient with IL without CCL group progressed to severe stenosis with unstable angina and need revascularization at 8years FU. RCA; right coronary artery, IL; intermediate lesion, CCL; combined culprit lesion, LAD; left descending artery, FU; follow up
