# Supplementary material for: Ten‐year clinical outcomes in patients with intermediate coronary stenosis according to the combined culprit lesion
Source: Clin Cardiol. 2021 Jun 16;44(8):1161–8. doi: 10.1002/clc.23668 (PMC8364722; doi:10.1002/clc.23668)
Supplement: Supplementary file 2 — Figure S2 xxxx [file CLC-44-1161-s001.pdf]

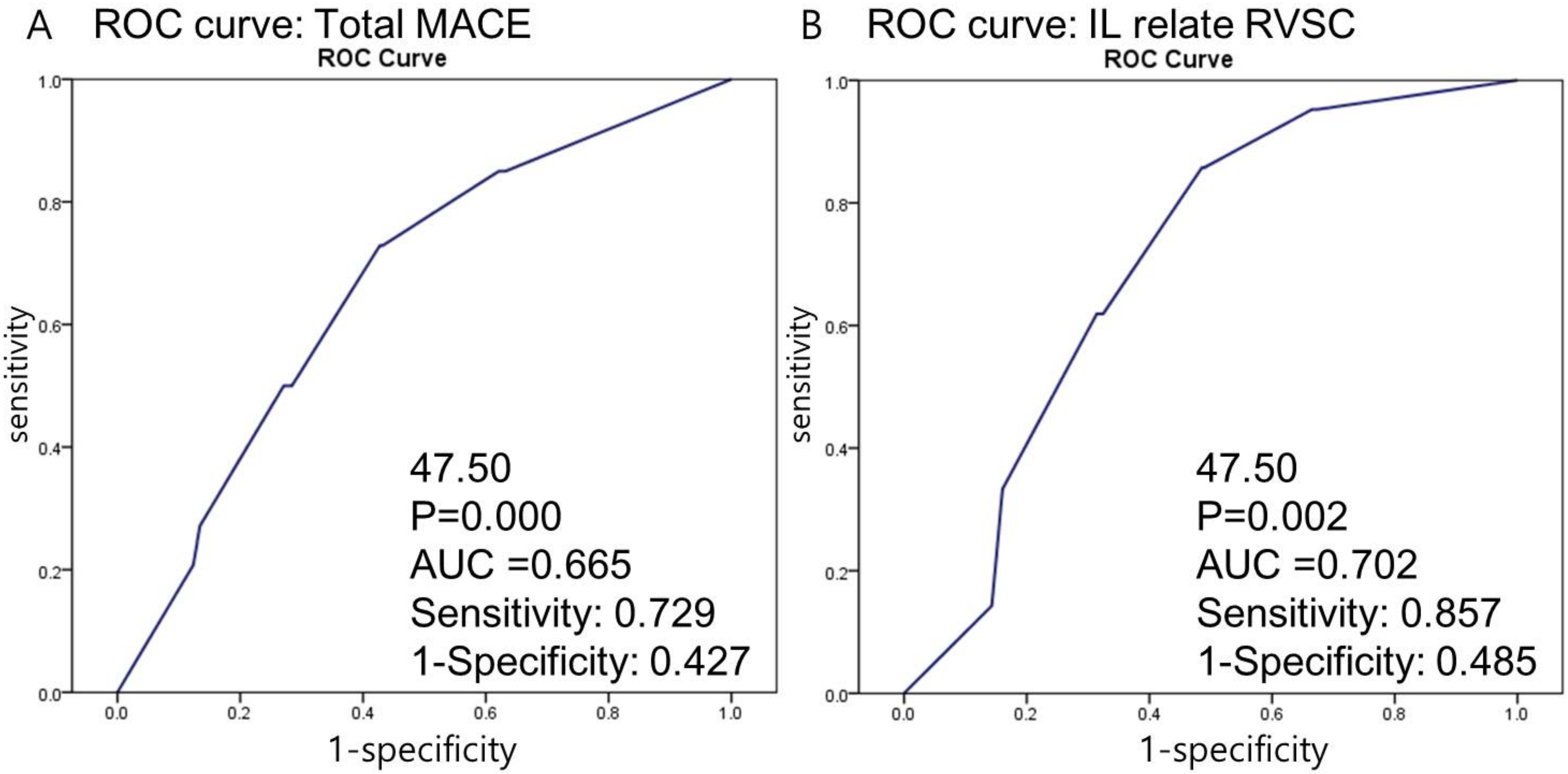

Supplementary figure 2. (A; left, B; right). ROC curves for IL percent diameter stenosis for Total MACE and IL relate RVSC. ROC; receiver operator characteristic, IL; intermediate lesion, MACE; major adverse cardiovascular event, RVSC; revascularization, AUC; area under curve
